# Trustworthiness assessment as an inclusion criterion for systematic reviews—What is the impact on results?

**DOI:** 10.1002/cesm.12037

**Published:** 2023-12-13

**Authors:** Jo Weeks, Anna Cuthbert, Zarko Alfirevic

**Affiliations:** ^1^ Cochrane Pregnancy and Childbirth Group University of Liverpool Liverpool UK

**Keywords:** Cochrane, research integrity, trustworthiness tool

## Abstract

**Background:**

There is increasing concern that a significant proportion of randomized controlled trials (RCTs) included in Cochrane reviews may not be trustworthy. Applying the Cochrane Pregnancy and Childbirth Trustworthiness Screening Tool (CPC‐TST) has already had a clinically important effect on several reviews published by the Cochrane Pregnancy and Childbirth Group.

**Objectives:**

We wanted to assess the impact of removing untrustworthy RCTs from already‐published Cochrane reviews on a defined clinical area (antenatal and postnatal nutritional interventions).

**Methods:**

We applied the tool to 18 Cochrane reviews (374 RCTs). The tool had four domains: (i) is the research governance trustworthy; (ii) are the baseline characteristics trustworthy; (iii) is the study feasible; (iv) are the results plausible? When additional information was needed, authors were contacted using a standard template. At least two attempts were made to contact the authors. At the end of the evaluation process each study was classified as: (i) included (YES to all questions); (ii) excluded (retracted study); or (iii) awaiting classification (any NO to the questions).

**Results:**

Ninety‐three out of 374 included studies (25%) were reclassified as “excluded” or “awaiting classification.” The number of included RCTs was reduced in 14 out of 18 reviews. Six reviews (33%) were judged to require updating because of important differences in the Summary of Findings tables (direction and size of effects and/or GRADE ratings), conclusions, implication for practice, and/or implication for research.

**Conclusions:**

Formal assessment of trustworthiness, and inclusion only of studies that satisfy prespecified criteria for trustworthiness, affect conclusions in a relatively large number of Cochrane reviews, with potentially important clinical implications for practice and research.

## 1 INTRODUCTION

Inclusion of untrustworthy and potentially fraudulent trials in meta‐analyses and clinical guidelines may have serious implications for healthcare decisions that are informed by highly influential systematic reviews, including Cochrane reviews. Despite the growing body of literature drawing attention to this issue, little action has been taken and large numbers of trials continue to be published that do not meet expected standards of conduct including lack of transparency, restricted access to data for external validation, and poor regulation and governance [1, 2, 3].

Current trial assessment tools used in systematic reviews (e.g., GRADE) are not designed to detect study aspects indicative of untrustworthy science, such as improbable numbers of eligible participants, or implausible features such as no participant dropouts during the course of a prolonged follow‐up phase. Although Cochrane as a whole does have a clearly outlined research integrity policy, there is no uniformly accepted process to define and deal with potentially untrustworthy studies. To address this, Cochrane Pregnancy and Childbirth introduced a trustworthiness screening tool [4], with a clearly defined process to include only studies with trusted data. This tool has already been used in several published review updates [5, 6]. As with any tool, it cannot guarantee to pick up all problems, or ensure that all included data are true.

The aim of this study was to assess the impact of assessment of trustworthiness of included trials on the results and conclusions of 18 Cochrane reviews of nutritional interventions during pregnancy and the postnatal period.

## 2 METHODS

This work was funded by the Children's Investment Fund Foundation, an independent philanthropic organization, who expressed particular interest in Cochrane Pregnancy and Childbirth's reviews tackling nutritional interventions.

### 2.1 Selection of the tool

We used a trustworthiness tool already developed for Cochrane reviews by the editorial team of the Pregnancy and Childbirth group and reviewed by the group's editorial board consisting of topic experts and consumer representatives [4]. The tool has subsequently been used by others; for instance Negrini et al. have used it with COPE guidelines to assess two papers published in their journal [7], and O'Connell et al. have used a modified form of the tool with the Cochrane Risk of Bias tool to check the papers of one author's published studies on spinal pain [8].

### 2.2 Selection of the reviews

We searched all published Cochrane reviews by our group, looking for reviews of all nutritional interventions during pregnancy and childbirth with at least 10 included studies and at least one Summary of Findings Table (SoF) applied in accordance with the Cochrane handbook [9]. We also looked for evidence that a potentially eligible review was included in at least one published guideline.

### 2.3 Application of the tool

One of the authors (JW) applied the Cochrane Pregnancy and Childbirth Trustworthiness Screening Tool to all 374 RCTs, consulting another coauthor (ZA) when decisions were not obvious. The tool had four domains, with questions to facilitate the screening process (Table 1).

**Table 1:** The four domains of the Cochrane pregnancy and childbirth trustworthiness screening tool.

Domain	Suggested questions to facilitate screening process
1. Research governance	Are there any retraction notices or expressions of concern listed on the Retraction Watch Database [10] relating to this study? Was the study prospectively registered (for those studies submitted in or after 2010)? If not, have the authors provided a plausible reason? When requested, did the trial authors provide/share the protocol and/or ethics approval letter? Did the trial authors engage in communication with Cochrane Pregnancy and Childbirth within the agreed timelines? Did the trial authors provide IPD data upon request? If not, was there a plausible reason?
2. Baseline characteristics	Is the study free from characteristics of the study participants that appear too similar, (e.g., distribution of the mean (SD) excessively narrow or excessively wide, as noted by Carlisle [11])?
3. Feasibility	Is the study free from characteristics that could be implausible (e.g., large numbers of women with a rare condition recruited from a single center within 12 months)? In cases with (close to) zero losses to follow‐up, is there a plausible explanation?
4. Results	Is the study free from results that could be implausible? (e.g., massive risk reduction for main outcomes with small sample size, evidence of copying.) Compared with other studies in the review, is the study free from very different results? (e.g., huge benefits with no complications or side‐effects, but others show that benefits are not so pronounced and there are complications.)

*Note*: See Appendix 2 for full details of the version of the CPC‐TST used.

The tool was further refined by the original developers to allow for historical changes in the expectations for research methods. Furthermore, the data from abstracts were only included if, in addition to the trustworthiness assessment, the study authors confirmed in writing that the data to be included in the review came from the final analysis and would not change (see Appendix 2 for full details). When additional information was needed, authors were contacted by emails based on a standard template. If an author's email address could not be found, their nearest Cochrane Center was contacted for help. A record of communications with authors was kept and at least two attempts were made to contact them over at least a 3‐month time period. The classification took into account the following criteria:

- Did the study authors engage in communication?
- Did the study authors provide data upon request? If not, was there a plausible reason?
- If requested, did the study authors provide/share the protocol and/or ethics approval letter?

At the end of the evaluation process each RCT was classified as:

- included: met all the tool criteria,
- excluded: the RCT was already retracted or had a publisher's expression of concern,
- awaiting classification: did not meet all the tool criteria.

### 2.4 Assessment of the tool's impact on reviews

For published reviews which included studies that were reclassified as “excluded” or “awaiting classification” after the tool was applied, SoF tables were recalculated and compared with the originals. The impact of the tool was quantified by calculating the following:

- the difference in the numbers of included studies in each review before and after the tool was applied,
- the difference in the direction and size of effects of SoF outcomes,
- any clinically important impact on conclusions, implications for practice, or implications for research.

## 3 RESULTS

We identified 18 Cochrane reviews that fulfilled our criteria for selection (Appendix 1). A total of 374 RCTs were included in these 18 Cochrane reviews.

### 3.1 Application of the tool

**Figure 1:**
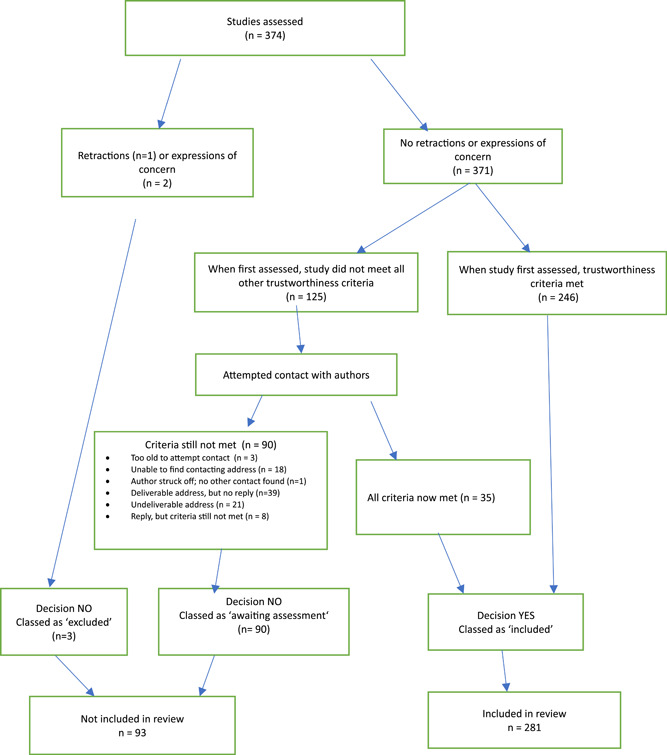
Flowchart showing the impact of the cochrane pregnancy and childbirth**—**trustworthiness screening Tool (CPC‐TST).

Out of 374 assessed RCTs, two‐thirds (246/374; 66%) satisfied all criteria when the tool was first applied (Figure 1). Out of the 128 RCTs that did not meet the criteria, three were because of retraction or expression of concern. When we tried to establish contact with the authors of all the remaining 125 studies that did not meet all criteria, 43 out of the 125 responded with further information, after which 35 were judged to be adequate (met all the criteria), and 8 still did not meet all the criteria because the additional information was not satisfactory. We were unable to establish any contact address for 18 out of the 125; for 39/125 we received no reply after one letter and one reminder (Figure 1).

### 3.2 Final inclusions/exclusions

At the end of the process, three‐quarters (281/374; 75%) of the RCTs remained as included. Three RCTs were excluded (one with a retraction and two with expressions of concern) and about a quarter (90/374; 24%) were placed in “awaiting classification.” See Figure 1 for full details.

### 3.3 Reasons for removal

For the 90 RCTs placed in “awaiting classification,” the issues were mainly in domain 1 (governance; *n* = 68) and domain 3 (feasibility; *n* = 65). No study was affected by domain 4 (results). Sixty‐three out of the 90 RCTs (70%) had issues in more than one domain. The main issues were over feasibility (for instance, less than 6 months between the end of the trial and its publication, without reasonable explanation) and over governance (for instance, no prospective trial registration for trials submitted in or after 2010). For the 27 RCTs for which there were issues in only one domain, the main reason was a lack of full information; this was usually when only an abstract or poster was available and the authors were unable to provide further data for full assessment. See Table 2 below.

**Table 2:** Trustworthiness issues: Why RCTs did not meet the tool criteria.

	Did not meet criteria (90 RCTs)^a^		
Domain	Trustworthiness issues (affecting a single domain) (27 RCTs)	Trustworthiness issues (affecting multiple domains) (63 RCTs)	Total issues
Governance	9	59	68
Baseline	1	37	38
Feasibility	7	58	65
Result comparison	0	0	0
Information	10	1	11

^a^ Three studies had been already excluded for retraction or expression of concern, so other domains were not assessed.

### 3.4 Assessment of the tool's impact on reviews

Fourteen out of the 18 reviews were affected by the process. For these 14, the number of RCTs affected varied between 5% and 70% of the total of RCTs in the review. The process affected the summaries of findings of 13 of these reviews—at least one of their SoFs showed differences in the direction/size of effects and/or GRADE assessments. One had unchanged SoFs despite one affected RCT (Appendix 1).

### 3.5 Clinical importance

For a third (6/18) of the reviews, the removal of affected RCTs led to changes to their SoFs which were considered clinically important. For seven of the 18 reviews, RCTs were removed but this led to changes in their SoFs which were considered not clinically important. Four of the 18 reviews, with 45 included RCTs, were unaffected by the process.

See Appendix 1 for full details and references for the 18 Cochrane reviews and the effect of the tool.

See Appendix 3 for before‐and‐after comparisons of SoFs for the six Cochrane reviews for which we judged the tool to have made a clinically important difference.

## 4 DISCUSSION

In our study, a quarter of the RCTs did not meet the trustworthiness criteria. Our findings are comparable to Carlisle's analysis of submissions to the journal *Anaesthesia*, for which he concluded that “the extent of false data was sufficient to invalidate the scientific value of a quarter of papers submitted” [12]. Importantly in our study, when only RCTs judged to be trustworthy were included, the number of included studies changed in 14 out of the 18 Cochrane reviews. For six of the 18 reviews, using the tool resulted in changes that we judged as clinically important. Our results support the general consensus on the seriousness of the problem of untrustworthy data. The widespread use of trustworthiness tools would therefore be likely to impact a large proportion of meta‐analyses and clinical guidelines, and thus patient outcomes.

No concerns were categorized under domain 4 (results). This was for two reasons. First, domain 4 asked whether there are obvious “outliers” compared to other RCTs, and there were none for the reviews that we assessed (as this question may be regarded as too subjective and open to biased “post hoc” assessments, particularly in relation to legitimate outliers, we suggest that if the RCT passes the trustworthiness tool criteria in all other respects, any outlying results should rather be investigated thoroughly by review authors under the RoB and GRADE assessments on the presumption of legitimacy). Secondly, the other question in domain 4 was about the feasibility of results in general. As this was already covered by domain 3 (feasibility) any issues here were categorized under domain 3 (such as when published *p*‐values for results were not recalculable). As the domains “governance” (domain 1) and “feasibility” (the criteria for the other three domains) would identify all trials affected by the tool either individually or in combination, we further suggest that these two domains could be used as a minimum standard for any future trustworthiness assessment.

Although many tools have been developed [13, 14, 15, 16, 17], none of them have been formally validated, as highlighted by Khan et al. [18] Some authors have called for formal validation as an essential prerequisite to implementation [17, 18, 19]. However, validation of a trustworthiness tool is conceptually different from evaluation of diagnostic tests. It is an *aide memoire* for peer‐reviewers, editors and systematic reviewers to check for obvious issues; importantly, we must accept that there may never be a gold standard against which tools can be evaluated for test accuracy. We acknowledge that some aspects of our tool, particularly those related to feasibility, are subjective and the questions are suggestions only, inviting authors to modify the tool depending on the clinical topic. This is practical and inevitable. Trustworthiness as a concept is more a spectrum than a yes/no binary. While the final assessment outcome is binary, the final decision will sometimes have elements of subjectivity. Similarly, specific trustworthiness criteria for inclusion in a systematic review might vary depending on the question posed. As long as the assessment decisions are unbiased and publicly available, subjectivity and modification of a tool in specific circumstances should not be a barrier to implementation.

Our tool was relatively straightforward to apply, but the whole process was quite time‐consuming, especially because of our insistence that authors should have the right to reply. It took time to write the individual queries; to find author addresses if they were not given in the publications, or if addresses given were no longer working; to conduct correspondence with the authors that replied; and to discuss, track, document, and archive the whole process. We allowed a 3‐month period for authors to respond, reaching out at least twice. We are conscious that this aspect of our methodological approach may prevent large‐scale implementation, but we believe that there is no real alternative if the process is to be unbiased and transparent. Given the inconsistencies in governance arrangements and ethical considerations globally, and the restrictions regarding published material imposed by some editors and publishers, one cannot rely solely on the information in the published materials.

It is important to stress that out of the 93 RCTs that were affected by the tool, only three did so because of an already‐published retraction or expression of concern, confirming that the bar for retraction is considerably higher than for trustworthiness assessment. The retraction rate for published papers, though growing, is only about eight in every 10,000 [12]. This is because the current emphasis is on someone (peer reviewer, journal editor, or systematic reviewer) having to prove that a submitted or published paper is flawed. For instance, the most common reasons for retraction in obstetrics and gynecology are plagiarism and data falsification [20]. This creates a bar that is far too high, and patients suffer as a consequence. We argue that the onus should be on researchers to answer any questions about their studies brought up by a trustworthiness test, passing the test by demonstrating in a clear and transparent way that the research they have put in the public domain is trustworthy. And if this requires a postpublication dialog with the research community, so be it. We acknowledge that listing RCTs that do not meet trustworthiness criteria in future Cochrane reviews as “awaiting assessment” will be seen by many as an interim measure only. Ideally, any such study should be eventually either retracted (excluded) or included, but such a simple binary approach is unlikely to be feasible. We are encouraged that the discussions regarding these issues are ongoing within the Cochrane community.

Although the concepts of research integrity assessment and validation of tests may not be controversial, as yet there is no consensus on the correct methodological approach. However, the tool that we used had several advantages. It has few domains, is relatively simple and quick to apply, and is a process that is transparent as long as the decisions are made publicly available. We argue that this is enough to start working with. It is important to stress that consensus‐based standards are an important part of improving the quality of tools, including this one; we look forward to further developments, and hope that this study goes towards increasing knowledge and agreement on the subject for all stakeholders.

## 5 CONCLUSION

A formal test of trustworthiness affected conclusions in a relatively large proportion of Cochrane reviews. This has potentially crucial clinical implications for clinical practice and research. The tool that we used is relatively simple and quick to apply. The whole process is transparent and reproducible although an element of subjectivity in any such assessments are inevitable. Any disagreements on the most appropriate tool, and lack of robust data on test accuracy, should not be used as an excuse to ignore the issue of research integrity of trials considered for inclusion in systematic reviews. We urgently call for all future Cochrane reviews to introduce some form of trustworthiness assessment, as there is no time to waste when it comes to patient safety.

## AUTHOR CONTRIBUTIONS

**Jo Weeks**: Contribution; methodology; data investigation; data curation; formal analysis; writing (original draft and review). **Anna Cuthbert**: Contribution; data curation; formal analysis; writing (review). **Zarko Alfirevic**: Contribution; conceptualization; funding acquisition; project administration; methodology; data curation; writing (original draft and review).

## CONFLICT OF INTEREST STATEMENT

Zarko Alfirevic is a member of the Editorial Board of the Cochrane Library. Jo Weeks and Anna Cuthbert declare no conflict of interest.

## PEER REVIEW

The peer review history for this article is available at https://www.webofscience.com/api/gateway/wos/peer-review/10.1002/cesm.12037.

## ETHICS STATEMENT

This work found that the removal of studies that did not fulfill prespecified trustworthiness criteria affected the conclusions of a third of the Cochrane reviews that we assessed.

## Data Availability

Data sharing, including correspondence with primary authors, is currently being discussed with the Cochrane Collaboration – it is hoped that all relevant documentation will shortly be available on the Cochrane website.

## APPENDIX 1 COCHRANE REVIEWS AND THE EFFECT OF THE COCHRANE PREGNANCY AND CHILDBIRTH TRUSTWORTHINESS SCREENING TOOL (CPC‐TST)

See Tables A1 and Table A2.

**Table A1:** Cochrane reviews with changes judged to be clinically important.^a^

Literature search date	Title, authors and date of Cochrane review	RCTs that were affected by the CPC‐TST or excluded for retraction/expression of concern	Pre‐tool RCTs Participants	Post‐tool RCTs Participants
**Cochrane reviews with clinically important changes in at least one published Summary of Findings (SoF) [6 reviews]**				
2014	1. Supplementation with long‐chain polyunsaturated fatty acids (LCPUFA) to breastfeeding mothers for improving child growth and development. Delgado‐Noguera et al. [21]	2 placed in “awaiting assessment”: Furuhjelm 2009 Jensen 2005	8 1567	6 1195
2014	2. Calcium supplementation (other than for preventing or treating hypertension) for improving pregnancy and infant outcomes. Buppasiri et al. [22]	5 placed in “awaiting assessment”: Ettinger 2009 Karandish 2003 Niromanesh 2001 Taherian 2002 Wanchu 2001	25 18,587	20 16,720
2015	3. Daily oral iron supplementation during pregnancy. Peña‐Rosas et al. [23]	11 placed in “awaiting assessment”: Corrigan 1936 Han 2001 Hankin 1963 Korkmaz 2014 Liu 2000 Liu 2012 Ma 2010 Ouladsahebmadarek 2011 Sun 2010 Ziaei 2007 Ziaei 2008	61 43,274	50 21,259
2015	4. Pyridoxine (vitamin B6) supplementation during pregnancy or labor for maternal and neonatal outcomes. Salam et al. [24]	1 placed in “awaiting assessment”: Hillman 1963	4 1646	3 114
2018	5. Vitamin D supplementation for women during pregnancy. Palacios et al. [25]	2 excluded and 19 placed in “awaiting assessment”: Asemi 2012 Asemi 2013 (excluded) Brooke 1980 Delvin 1986 Kaur 1991 Li 2000a Mallet 1986 Marya 1987 Marya 1988 Mazurkevich 2013 Mirghafourvand 2013 Nagshineh 2016 Sabet 2012 Samimi 2016 (excluded) Samimi 2017 Sasan 2017 Shahgheibi 2016 Singh 2015 Taherian 2002 Tehrani 2014 Vaziri 2016	30 7033	9 3721
2018	6. Regimens of vitamin D supplementation for women during pregnancy. Palacios et al. [26]	1 excluded and 11 placed in “awaiting assessment”: Bhatia 2012 Das 2010 de Menibus 1984. Hashemipour 2014 Kalra 2012 Karamali 2015 (excluded) Mallet 1986 Marya 1981 Mir 2016 Mojibian 2015 Shakiba 2013 Thiele 2014	30 7289	18 5846

^a^ For each of these six reviews, please see Appendix 3 for before‐and‐after comparisons of their Summary of Findings.

**Table A2:** Cochrane reviews with changes thought not to be clinically important.

Literature search date	Title, authors, and date of Cochrane review	RCTs that were affected by the CPC‐TST	Pretool RCTs Participants	Posttool RCTs Participants
**RCTs removed, at least one SoF changed, but no clinically important difference [7 reviews]**				
2015	7. Vitamin C supplementation in pregnancy. Rumbold et al. [27]	10 placed in “awaiting assessment”: Beazely 2005 Borna 2005 Ghomian 2013 Gungorduk 2014 Hans 2010 Huria 2010 Kalpdev 2011 Nasrolahi 2006 Rivas 2000 Sikkema 2002	29 24300	19 22273
2015	8. Intermittent oral iron supplementation during pregnancy. Peña‐Rosas et al. [28]	10 placed in “awaiting assessment”: Alaoddolehei 2013 Bhatla 2009 Bouzari 2011 Hashim 2012 Quintero 2004 Rukhsana 2006 Singh 2001 Viteri 2012 Yekta 2011 Yu 1998	27 5490	17 4247
2015	9. Treatment for women with postpartum iron deficiency anemia. Markova et al. [29]	3 placed in “awaiting assessment”: Guerra 2012 Mumtaz 2011 Verma 2011	22 2858	19 2442
2015	10. Vitamin E supplementation in pregnancy. Rumbold et al. [30]	9 placed in “awaiting assessment”: Beazely 2005 Borna 2005 Gungorduk 2014 Huria 2010 Kalpdev 2011 Nasrolahi 2006 Rivas 2000 Sawhney 2000 Shahraki 2006	21 22,129	12 20,492
2016	11. Iodine supplementation for women during the preconception, pregnancy and postpartum period. Harding et al. [31]	2 placed in “awaiting assessment”: Glinoer 1993 Liesenkotter 1996	14 >2700	12 228 removed
2016	12. Vitamin A supplementation for postpartum women. Oliveira et al. [32]	3 placed in “awaiting assessment”: Bhaskharam 2000 Venkatarao 1996 Vinutha 2000	14 25,758	11 24,638
2020	13. Zinc supplementation for improving pregnancy and infant outcome. Carducci et al. [33]	3 placed in “awaiting assessment”: Bangladesh 2016 China 2001 Iran 2010	25 >17,000	22 312 removed
**RCTs removed, no difference to the SoF(s). [1 review]**				
2015	14. Vitamin A supplementation during pregnancy for maternal and newborn outcomes. McCauley et al. [34]	1 placed in “awaiting assessment”: Sun 2010	19 >310,000	18 186 removed
**No RCTs removed. [4 reviews]**				
2015	15. Multiple micronutrient powders for home (point‐of‐use) fortification of foods in pregnant women. Suchdev et al. [35]		2 1172	2 1172
2015	16. Effects and safety of periconceptional oral folate supplementation for preventing birth defects. De‐Regil et al. [36]		5 7391	5 7391
2015	17. Antenatal dietary education and supplementation to increase energy and protein intake. Ota et. al. [37]		17 9030	17 9030
2018	18. Multiple‐micronutrient supplementation for women during pregnancy. Keats et al. [38]		21 141,849	21 141,849

## APPENDIX 2 THE COCHRANE PREGNANCY AND CHILDBIRTH TRUSTWORTHINESS SCREENING TOOL (CPC‐TST)

**N.B. This tool is a modified version of a previous version of the published Cochrane Pregnancy and Childbirth Group Trustworthiness Screening Tool** [4].

**Domain 1**—**Research governance**
  o Are there any retraction notices or expressions of concern listed on the Retraction Watch Database [10] relating to this study?
  o Was the study prospectively registered (for those studies submitted during or after 2010)? If not, have the authors provided a plausible reason?
  o When requested, did the trial authors provide/share the protocol and/or ethics approval letter?
  o Did the trial authors engage in communication with Cochrane Pregnancy and Childbirth within the agreed timelines?
  o Did the trial authors provide IPD data upon request? If not, was there a plausible reason?

**Domain 2**—**Baseline characteristics**
  o Is the study free from characteristics of the study participants that appear too similar, (e.g., distribution of the mean (SD) excessively narrow or excessively wide, as noted by Carlisle [11])?

**Domain 3**—**Feasibility**
  o Is the study free from characteristics that could be implausible (e.g., large numbers of women with a rare condition recruited from a single center within 12 months)?
  o In cases with (close to) zero losses to follow‐up, is there a plausible explanation?

**Domain 4**—**Results**
  o Is the study free from results that could be implausible? (e.g., massive risk reduction for main outcomes with small sample size, evidence of copying.)
  o Compared with other studies in the review, is the study free from very different results? (e.g., huge benefits with no complications or side‐effects, but others show that benefits are not so pronounced and there are complications.)

**Cases of insufficient data**

Data from abstracts or posters were only included if, in addition to the trustworthiness assessment, the study authors confirmed in writing that the data to be included in the review came from the final analysis and would not change.

**Allowing for historical changes in methodological/reporting expectations**

Studies submitted before 1990 were judged to have passed the tool criteria even if study dates, ethics approval, clear description of randomization and blinding process and dropout rates were not explicitly stated.

For studies submitted before 2010, mention of the trial registration was not required and either the study dates or the ethics/consent information could be missing from the writeup.

For studies submitted before 1980, we did not try to contact the authors because of the age of the publications and the age of the authors; rather, trustworthiness/GRADE decisions were made on what information was available in the public domain.

## APPENDIX 3 COMPARISONS OF SUMMARY OF FINDINGS: THE SIX REVIEWS FOR WHICH WE JUDGED THERE TO BE A CLINICALLY IMPORTANT DIFFERENCE IN SOFS AFTER THE CPC‐TST WAS APPLIED IN THE CHILDREN'S INVESTMENT FOUNDATION FUND (CIFF) STUDY

**Yellow highlights**: updates show exactly the same result.

**Orange highlights**: updates show very similar result.

**Aqua highlights**: updates show a different result.

Screenshot 1: Delgado‐Noguera MF, Calvache JA, Bonfill Cosp X, Kotanidou EP, Galli‐Tsinopoulou A. Supplementation with long chain polyunsaturated fatty acids (LCPUFA) to breastfeeding mothers for improving child growth and development. *Cochrane Database of Systematic Reviews* 2015, Issue 7 [21].

**Original Cochrane review conclusions**:

*Based on the available evidence, LCPUFA supplementation did not appear to improve children's neurodevelopment, visual acuity, or growth. In child attention at 5 years of age, weak evidence was found (one study) favouring the supplementation. Currently, there is inconclusive evidence to support or refute the practice of giving LCPUFA supplementation to breastfeeding mothers to improve neurodevelopment or visual acuity*.

**Our findings on SOF similarities after the tool was applied in our CIFF study update**:
  • For child weight the results were similar following the update; the 95% CI narrowed from (0.49 g lower to 0.74 g higher) to (0.8 g lower to 0.81 g higher), still crossing the line of no effect (orange highlights); and the GRADE certainty remained *moderate* (yellow highlights). This means that the effect of the current LCPUFA supplementation for breastfeeding mothers on child weight probably makes little or no difference.

**Our findings on SOF differences**:
  • For language development, the 95% confidence interval (CI) remained across the line of no effect; but the GRADE certainty changed from *moderate* to *very low*. This means that the effect of the current LCPUFA supplementation for breastfeeding mothers on infant language development, rather than probably making little or no difference, is uncertain (aqua highlights).
  • For intelligence or problem‐solving ability, the 95% CI remained across the line of no effect; but the GRADE certainty changed from *low* to *very low*. This means that the effect of the current LCPUFA supplementation for breastfeeding mothers on infant intelligence or problem‐solving ability, rather than maybe making little or no difference, is uncertain (aqua highlights).
  • For psychomotor development, child attention, and child visual acuity, the 95% CIs changed from crossing the line of no effect, and GRADE certainty *low*, to no studies reported. This means that the effect of the current LCPUFA supplementation for breastfeeding mothers on psychomotor development, child attention, and child visual acuity, rather than maybe making little or no difference, is unreported (aqua highlights).

**Our conclusions are therefore**: (changes highlighted in yellow)

*Based on the available evidence, the LCPUFA supplementation probably makes little or no difference to child weight ‐long term beyond 24 months (moderate certainty of evidence). We cannot be certain of the effect of LCPUFA supplementation on language development beyond 24 months, intelligence, or problem‐solving ability beyond 24 months (very low certainty of evidence). No trials were reported on the remaining three outcomes. Therefore, there is currently either no evidence or very little evidence to support or refute the practice of giving LCPUFA supplementation to breastfeeding mothers to improve neurodevelopment or visual acuity*.

**Summary**: Cochrane review update needed, as the review outcomes and certainty of evidence have changed from having mostly inconclusive outcomes with moderate/low certainty of evidence, to three outcomes with moderate/very low certainty of evidence and three empty outcomes. This has changed the review's conclusions.

**Yellow highlights**: updates show exactly the same result.

**Orange highlights**: updates show very similar result.

**Aqua highlights**: updates show a different result.

Screenshot 1: Buppasiri P, Lumbiganon P, Thinkhamrop J, Ngamjarus C, Laopaiboon M, Medley N. Calcium supplementation (other than for preventing or treating hypertension) for improving pregnancy and infant outcomes. *Cochrane Database of Systematic Reviews* 2015, Issue 2 [22].

**Original Cochrane review conclusions**:

*This review indicates that there are no clear additional benefits to calcium supplementation in prevention of preterm birth or low infant birthweight. While there was a statistically significant difference of 56 g identified in mean infant birthweight, there was significant heterogeneity identified, and the clinical significance of this difference is uncertain*.

**Our findings on SOF similarities after the tool was applied in our CIFF study update**:
  • For low birthweight (<2500 g), the effect estimate and GRADE assessment remained the same (yellow highlights).
  • For preterm birth before 34 weeks' gestation, the effect estimate was similar (orange highlights); and the GRADE assessment remained moderate (yellow highlights).

**Our findings on SOF differences**:
  • For preterm birth before 37 weeks, the 95% CI narrowed very slightly from (0.7–1.05) to (0.65–0.99), no longer crossing the line of no effect; and the GRADE certainty changed from *moderate* to *high*. This means that the effect of the current calcium supplementation on the risk of preterm birth before 37 weeks, rather than probably making little or no difference, has a positive effect (aqua highlights), when compared with placebo or no treatment.

**N.B. For preterm birth before 37 weeks' gestation, the authors removed the same two trials as were removed in the CIFF update, as part of a sensitivity analysis because of unclear risk of bias. See screenshot below from the Cochrane review.**

We also conducted sensitivity analyses by removing two included trials (Taherian, 2002; Wanchu, 2001) whose allocation of concealment was unclear. The results then favored treatment with calcium supplementation (risk ratio [RR], 0.80, 95% CI, 0.65–0.99; 11 trials, 15,379 women; random‐effects model; Analysis 1.1). There was significant heterogeneity for this outcome (Tau^2^ = 0.04; Chi^2^ = 20.46, *df* = 9 (*p* = 0.02); *I* ^2^ = 56%).

**Our conclusions are therefore**: (changes highlighted in yellow)

*This review indicates that there is high certainty that calcium supplementation reduces preterm birth before 37 weeks. There are no clear additional benefits to calcium supplementation in preterm birth before 34 weeks or prevention of low infant birthweight*.

**Summary**: Cochrane review update needed as the conclusions are different.

**Yellow highlights**: updates show exactly the same result.

**Orange highlights**: updates show very similar result.

**Aqua highlights**: updates show a different result.

Original screenshot: Peña‐Rosas JP, De‐Regil LM, Garcia‐Casal MN, Dowswell T. Daily oral iron supplementation during pregnancy. Cochrane Database of Systematic Reviews 2015a, Issue 7 [23].

**Yellow highlights**: updates show exactly the same result.

**Orange highlights**: updates show very similar result.

**Aqua highlights**: updates show a different result.

Original screenshot: Peña‐Rosas JP, De‐Regil LM, Garcia‐Casal MN, Dowswell T. Daily oral iron supplementation during pregnancy. *Cochrane Database of Systematic Reviews* 2015a, Issue 7 [23].

**Original Cochrane review conclusions**:

*Supplementation reduces the risk of maternal anemia and iron deficiency in pregnancy but the positive effect on other maternal and infant outcomes is less clear. Implementation of iron supplementation recommendations may produce heterogeneous results depending on the populations’ background risk for low birthweight and anemia, as well as the level of adherence to the intervention*.

**Our findings on SOF similarities after the tool was applied in our CIFF study update**:
  • There was no change to the data for “Any supplements containing iron and folic acid compared with same supplements without iron nor folic acid (no iron nor folic acid or placebo)—maternal and infant outcomes” so the SOF table for this comparison is not shown here.
  • For SOF 1 ([Infant outcomes] Any supplements containing iron compared with the same supplements without iron or no treatment/placebo (no iron or placebo) for pregnant women), birthweight, preterm birth (less than 37 weeks of gestation), neonatal death, and congenital anomalies had similar effect estimates (orange highlights); and GRADE assessments for neonatal death, and congenital anomalies stayed the same (yellow highlights).
  • For SOF 2 ([Maternal outcomes] Any supplements containing iron compared with the same supplements without iron or no treatment/placebo (no iron or placebo) for pregnant women), maternal iron deficiency at term, and maternal severe anemia at any time during second and third trimester had the same effect estimates and GRADE assessments (yellow highlights). Maternal anemia at term, and side effects had similar effect estimates (orange highlights), and GRADE assessments stayed the same (yellow highlights). GRADE assessment for maternal death also stayed the same.

**Our findings on differences to SOF 1 ([Infant outcomes] Any supplements containing iron compared with the same supplements without iron or no treatment/placebo [no iron or placebo] for pregnant women) after the update**:
  • For low birthweight (less than 2500 g), the 95% CI changed from (0.69–1.03) to (0.59–0.97); and the GRADE certainty changed from *low* to *moderate*. This means that the effect of the current iron supplementation on low birthweight, rather than possibly making little or no difference, probably has a positive effect (aqua highlights).
  • For birthweight, and preterm birth (less than 37 weeks of gestation), the effect estimates remained crossing the line of no effect, and the GRADE assessments changed from *moderate* to *low*.

**Our findings on differences to SOF 2 ([Maternal outcomes] Any supplements containing iron compared with the same supplements without iron or no treatment/placebo [no iron or placebo] for pregnant women) after the update**:
  • For maternal death, the effect estimate changed from RR 0.33 (95% CI: 0.01–8.19) to not estimable (no events) (blue highlights). The GRADE certainty remained *very low*; this means that the current iron supplementation on the risk of maternal death is uncertain.
  • For infection during pregnancy, the effect estimate changed from RR 1.21 (95% CI: 0.33–4.46), and GRADE certainty *low*, to no studies reported. This means that the current iron supplementation on the risk of infection during pregnancy, rather than maybe having little or no effect, is unreported (blue highlights).

**Our conclusions are therefore**: (changes highlighted in yellow)

*Supplementation may reduce the risk of maternal anemia and iron deficiency in pregnancy, and probably slightly reduces the incidence of low birthweight (less than 2500 g), but the positive effect on other maternal and infant outcomes is less clear. Implementation of iron supplementation recommendations may produce heterogeneous results depending on the populations’ background risk for low birthweight and anemia, as well as the level of adherence to the intervention*.

**Summary**: Removing the trials during the update has shown a probable positive effect on low birthweight (less than 2500 g); a Cochrane update is needed.

**Yellow highlights**: updates show exactly the same result.

**Orange highlights**: updates show very similar result.

**Aqua highlights**: updates show a different result.

Original screenshot: Salam RA, Zuberi NF, Bhutta ZA. Pyridoxine (vitamin B6) supplementation during pregnancy or labor for maternal and neonatal outcomes. *Cochrane Database of Systematic Reviews* 2015, Issue 6 [24].

**Cochrane review conclusions**:

*There were few trials, reporting few clinical outcomes and mostly with unclear trial methodology and inadequate follow‐up. There is not enough evidence to detect clinical benefits of vitamin B6 supplementation in pregnancy and/or labor other than one trial suggesting protection against dental decay. Future trials assessing this and other outcomes such as orofacial clefts, cardiovascular malformations, neurological development, preterm birth, pre‐eclampsia, and adverse events are required*.

**Our findings on similarities after the tool was applied in our CIFF study update**:
  • Preterm birth (before 37 weeks' gestation) and low birthweight remained unreported (yellow highlights).
  • For eclampsia‐ antenatal oral pyridoxine versus control, the effect estimate remained not estimable (no events) (yellow highlights).

**Our findings on differences**:
  • For dental decay—antenatal oral pyridoxine versus control, the effect estimate changed from RR 0.84 (95% CI: 0.71–0.98), to no studies reported. This means that the current antenatal oral pyridoxine supplementation on the risk of dental decay, rather than being possibly positive, is unreported (aqua highlights).
  • For dental decay—antenatal pyridoxine lozenges versus control, the effect estimate changed from RR 0.68 (95% CI: 0.56–0.83), to no studies reported. This means that the current antenatal pyridoxine lozenges supplementation on the risk of dental decay, rather than being possibly positive, is unreported (aqua highlights).
  • For eclampsia—antenatal oral pyridoxine versus control, the effect estimate remained not estimable (no events) (yellow highlights). The original Cochrane review did not GRADE this outcome. Following the CIFF update, the GRADE certainty is *very low*; this means that the current antenatal oral pyridoxine supplementation on the risk of eclampsia is uncertain (blue highlights).
  • For pre‐eclampsia—antenatal oral pyridoxine versus control, the effect estimate changed from RR 1.71 (95% CI: 0.85–3.45), to not estimable (no events); and the GRADE certainty changed from *low* to *very low*. This means that the current antenatal oral pyridoxine supplementation on the risk of pre‐eclampsia, rather than possibly having little or no effect, is uncertain (blue highlights).
  • For pre‐eclampsia—antenatal pyridoxine lozenges versus control, the effect estimate changed from RR 1.43 (95% CI: 0.64–3.22), to no studies reported. This means that the current antenatal pyridoxine lozenges supplementation on the risk of pre‐eclampsia, rather than possibly having little or no effect, is unreported (blue highlights).

**Our conclusions** are therefore now: (changes highlighted in yellow)

*There were few trials, reporting few clinical outcomes and mostly with unclear trial methodology and inadequate follow‐up. There is not enough evidence to detect clinical benefits of vitamin B6 supplementation in pregnancy and/or labor. Future trials assessing this and other outcomes such as dental decay, orofacial clefts, cardiovascular malformations, neurological development, preterm birth, pre‐eclampsia, and adverse events are required*.

**Summary**: The updated SOF is largely “empty” but this does not change much as the conclusions from the original Cochrane review reflect this. However, the original conclusion mentioned protection against dental decay and this should no longer be the case, so a **Cochrane review update is perhaps needed**.

**Yellow highlights**: updates show exactly the same result.

**Orange highlights**: updates show very similar result.

**Aqua highlights**: updates show a different result.

Original screenshot: Palacios C, Kostiuk LK, Peña‐Rosas JP. Vitamin D supplementation for women during pregnancy. *Cochrane Database of Systematic Reviews* 2019a, Issue 7 [25].

**Yellow highlights**: updates show exactly the same result.

**Orange highlights**: updates show very similar result.

**Aqua highlights**: updates show a different result.

Original screenshot: Palacios C, Kostiuk LK, Peña‐Rosas JP. Vitamin D supplementation for women during pregnancy. *Cochrane Database of Systematic Reviews* 2019a, Issue 7 [25].

**Original Cochrane review conclusions**:

*We included 30 trials (7033 women) across three separate comparisons. Our GRADE assessments ranged from moderate to very low, with downgrading decisions based on limitations in study design, imprecision, and indirectness*.

*Supplementing pregnant women with vitamin D alone probably reduces the risk of pre‐eclampsia, gestational diabetes, low birthweight and may reduce the risk of severe postpartum hemorrhage. It may make little or no difference in the risk of having a preterm birth* <*37 weeks' gestation. Supplementing pregnant women with vitamin D and calcium probably reduces the risk of pre‐eclampsia but may increase the risk of preterm births* <*37 weeks (these findings warrant further research). Supplementing pregnant women with vitamin D and other nutrients may make little or no difference in the risk of preterm birth* <*37 weeks' gestation or low birthweight (less than 2500 g). Additional rigorous high‐quality and larger randomized trials are required to evaluate the effects of vitamin D supplementation in pregnancy, particularly in relation to the risk of maternal adverse events*.

**Our findings on SOF similarities after the tool was applied in our Children's Investment Foundation Fund (CIFF) study update**:
  • There was no change to the data for “Vitamin D + calcium + other vitamins and minerals compared to calcium + other vitamins and minerals (but no vitamin D) for pregnancy and neonatal health outcomes” so the SOF table for this comparison is not shown here.
  • For SOF 1 (Vitamin D vs. placebo or no intervention for pregnancy and neonatal outcomes) three maternal adverse events outcomes had the same effect estimates (yellow highlights); and GRADE assessments for two of these outcomes stayed the same (yellow highlights). Preterm birth (less than 37 weeks' gestation) had a very similar effect estimate (orange highlights).
  • For SOF 2 (Vitamin D + calcium vs. placebo or no intervention for pregnancy and neonatal outcomes) the only three outcomes remaining unchanged by the CIFF update were the maternal adverse events outcomes which remained unreported (yellow highlights). For low birthweight (less than 2500 g), the GRADE certainty remained *very low* (blue highlights).

**Our findings on differences to SOF 1 (vitamin D alone) after the update**:
  • For pre‐eclampsia, the 95% CI widened from (0.30–0.79) to (0.21–1.33), across the line of no effect; and the GRADE certainty changed from *moderate* to *very low*. This means that the effect of the current Vitamin D supplementation on the risk of pre‐eclampsia, rather than being probably positive, is uncertain (aqua highlights).
  • For gestational diabetes, the 95% CI widened from (0.27–0.97) to (0.03–8.28), across the line of no effect; and the GRADE certainty changed from *moderate* to *very low*. This means that the effect of current Vitamin D supplementation on the risk of gestational diabetes, rather than being probably positive, is uncertain (aqua highlights).
  • For low birthweight (less than 2500 g), the 95% CI widened from (0.35–0.87) to (0.44–1.08), across the line of no effect; and the GRADE certainty changed from *moderate* to *low*. This means that the effect of current Vitamin D supplementation on the risk of low birthweight, rather than being probably positive, may make little or no difference (aqua highlights).
  • For hypercalcaemia and for preterm birth (less than 37 weeks' gestation), the GRADE certainty changed from *low* to *very low* (blue highlights). This means that the current Vitamin D supplementation on the risk of hypercalcaemia, and preterm birth (less than 37 weeks' gestation), is uncertain.

**Our findings on differences to SOF 2 (vitamin D and calcium) after the update**:
  • For pre‐eclampsia, the effect estimate changed from RR 0.50 (95% CI: 0.32–0.78), and GRADE certainty *moderate*, to no studies reported. This means that the current Vitamin D + calcium supplementation on the risk of pre‐eclampsia, rather than being probably positive, is unreported (aqua highlights).
  • For gestational diabetes, the effect estimate changed from RR 0.33 (95% CI: 0.01–7.84), and GRADE certainty *very low*, to no studies reported. This means that the current Vitamin D + calcium supplementation on the risk of gestational diabetes, rather than being uncertain, is unreported (aqua highlights).
  • For preterm birth (less than 37 weeks), the effect estimate changed from RR 1.52 (95% CI: 1.01–2.28) to not estimable (no events); and the GRADE certainty changed from *low* to *very low*. This means that the current Vitamin D + calcium supplementation on the risk of preterm birth <37 weeks, rather than being possibly negative, is uncertain (aqua highlights).

**Our conclusions are therefore**: (changes highlighted in yellow)

*We included 7 trials (3721 women) across three separate comparisons. Our GRADE assessments ranged from low to very low, with downgrading decisions based on limitations in study design, imprecision, and indirectness*.

*Supplementing pregnant women with vitamin D alone may reduce the risk of severe postpartum hemorrhage (low certainty evidence). It may make little or no difference to low birthweight (low certainty evidence), but we are uncertain of its effect on pre‐eclampsia, gestational diabetes, and preterm birth* <*37 weeks' gestation (very low certainty evidence)*.

*Supplementing pregnant women with vitamin D and calcium has an uncertain effect on the risk of low birthweight, and preterm births* <*37 weeks (very low certainty evidence). Pre‐eclampsia, gestational diabetes, and maternal adverse events were not reported*.

*Supplementing pregnant women with vitamin D and other nutrients may make little or no difference in the risk of preterm birth* <*37 weeks' gestation or low birthweight (less than 2500 g). Additional rigorous high‐quality and larger randomized trials are required to evaluate the effects of vitamin D supplementation in pregnancy, particularly in relation to the risk of maternal adverse events*.

**Summary**: Cochrane review update needed as the conclusions are different.

**Yellow highlights**: updates show exactly the same result.

**Orange highlights**: updates show very similar result.

**Aqua highlights**: updates show a different result.

Original screenshot: Palacios C, Trak‐Fellermeier MA, Martinez RX, Lopez‐Perez L, Lips P, Salisi JA, John JC, Peña‐Rosas JP. Regimens of vitamin D supplementation for women during pregnancy. *Cochrane Database of Systematic Reviews* 2019b, Issue 10 [26].

Original screenshot: Palacios C, Trak‐Fellermeier MA, Martinez RX, Lopez‐Perez L, Lips P, Salisi JA, John JC, Peña‐Rosas JP. Regimens of vitamin D supplementation for women during pregnancy. *Cochrane Database of Systematic Reviews* 2019b, Issue 10 [26].

**Yellow highlights**: updates show exactly the same result.

**Orange highlights**: updates show very similar result.

**Aqua highlights**: updates show a different result.

Original screenshot: Palacios C, Trak‐Fellermeier MA, Martinez RX, Lopez‐Perez L, Lips P, Salisi JA, John JC, Peña‐Rosas JP. Regimens of vitamin D supplementation for women during pregnancy. *Cochrane Database of Systematic Reviews* 2019b, Issue 10 [26].

**Original Cochrane review conclusions**:

*Supplementing pregnant women with more than the current vitamin D recommendation may reduce the risk of gestational diabetes; however, it may make little or no difference to the risk of pre‐eclampsia, preterm birth, and low birthweight. Supplementing pregnant women with more than the current upper limit for vitamin D seems not to increase the risk of the outcomes evaluated. In general, the GRADE was considered low certainty for most of the primary outcomes due to serious risk of bias and imprecision of results. With respect to safety, it appears that vitamin D supplementation is a safe intervention during pregnancy, although the parameters used to determine this were either not reported or not consistent between trials. Future trials should be consistent in their reports of adverse events. There are 16 ongoing trials that when published, will increase the body of knowledge*.

**Our findings on SOF similarities after the tool was applied in our CIFF study update**:
  • For SOF 1 (A dose of vitamin D 601 IU or higher compared to 600 IU or lower alone with other nutrients for women during pregnancy), pre‐eclampsia, preterm birth, and low birthweight had similar effect estimates (orange highlights).
  • For SOF 2 (A dose of vitamin D 4000 IU/d or more compared to 3999 IU/d or less alone with any other nutrient for women during pregnancy), gestational diabetes had the same effect estimate; and the GRADE assessment remained the same (yellow highlights). Pre‐eclampsia, preterm birth, and low birthweight had similar effect estimates (orange highlights).

**Our findings on differences to SOF 1 (dose of vitamin D 601 IU or higher compared to 600 IU or lower alone or with other nutrients) after the update**:
  • For gestational diabetes, the 95% CI widened from (0.34–0.86) to (0.28–1.23), across the line of no effect; and the GRADE certainty changed from *moderate* to *low*. This means that the effect of a 601 IU dose or higher of vitamin D supplementation on the risk of gestational diabetes, rather than being probably positive, possibly makes little or no difference (aqua highlights), when compared with a lower dose of 600 IU or below.
  • For pre‐eclampsia and preterm birth, the GRADE certainty changed from *low* to *moderate*. This means that the effect of a 601 IU dose or higher of vitamin D supplementation on the risk of pre‐eclampsia, and preterm birth, rather than possibly having little or no effect, probably has little or no effect (blue highlights), when compared with a lower dose of 600 IU or below.
  • For low birthweight, the GRADE certainty changed from *very low* to *moderate*. This means that the effect of a 601 IU dose or higher of vitamin D supplementation on the risk of low birthweight, rather than being uncertain, probably has little or no effect (blue highlights), when compared with a lower dose of 600 IU or below.

**Our findings on differences to SOF 2 (dose of vitamin D 4000 IU/d or more compared to 3999 IU/d or less alone or with any other nutrient) after the update**:
  • For pre‐eclampsia, preterm birth, and low birthweight, the GRADE certainty changed from *low* to *moderate*. This means that the effect of a 4000 IU/d dose or more of vitamin D supplementation on the risk of pre‐eclampsia, preterm birth, and low birthweight rather than possibly having little or no effect, probably has little or no effect (blue highlights), when compared with a lower dose of 3999 IU/d or less.

**Our conclusions are therefore**: (changes highlighted in yellow)

*Supplementing pregnant women with more than the current vitamin D recommendation may make little or no difference to the risk of gestational diabetes, pre‐eclampsia, preterm birth, and low birthweight. Supplementing pregnant women with more than the current upper limit for vitamin D seems not to increase the risk of the outcomes evaluated. In general, the GRADE was considered moderate certainty for most of the primary outcomes. With respect to safety, it appears that vitamin D supplementation is a safe intervention during pregnancy, although the parameters used to determine this were either not reported or not consistent between trials. Future trials should be consistent in their reports of adverse events. There are 16 ongoing trials that when published, will increase the body of knowledge*.

**Summary**: Cochrane review update needed as the conclusions are different.
